# Divergent rhizosphere microbial necromass dynamics between two plant species in response to water addition in a semi-arid region

**DOI:** 10.1038/s41598-026-52433-7

**Published:** 2026-05-10

**Authors:** Yang Yang, Jinchen Yang, Zhichao Yu, Jinghao Sun, Jin Zhang, Chuang Zhang, Yanan Wang, Yanqing He, Hongwei Pei

**Affiliations:** 1 Department of Municipal and Environmental Engineering, Hebei University of Architecture, Zhangjiakou, 075000 China; 2 Institute of Geographical Sciences, Henan Academy of Sciences, Zhengzhou, 450052 China; 3 Zhangjiakou Construction Engineering Quality Testing and Inspection Center Co., Ltd, Zhangjiakou, 075051 China; 4 Hebei Key Laboratory of Water Quality Engineering and Comprehensive Utilization of Water Resources, Zhangjiakou, 075000 China

**Keywords:** Soil moisture change, Microbial necromass carbon, Nutrient availability, Rhizosphere soil, Semi-arid area, Ecology, Ecology, Environmental sciences, Microbiology, Plant sciences

## Abstract

Microbial necromass carbon influences soil organic carbon (SOC) persistence, yet its response to soil moisture change in semi-arid rhizospheres remains unclear. *Avena sativa* and *Leymus chinensis*, which are widely cultivated in semi-arid areas, were selected for a three-month indoor pot cultivation experiment. The responses of soil microbial necromass carbon to water addition and the key drivers in the rhizosphere soil of these plants were identified. The results demonstrated that water addition significantly decreased the concentrations of bacterial necromass carbon (BNC), fungal necromass carbon (FNC), and SOC in the *Avena sativa* rhizosphere, but increased them in the *Leymus chinensis* rhizosphere. However, water addition consistently reduced the contribution of microbial necromass carbon to SOC across both rhizosphere soils. The FNC accounted for a larger proportion of SOC than BNC, but the dynamic of SOC was more closely associated with the change in BNC. Both BNC and FNC showed significant correlations with microbial properties (gene abundances and enzyme activities). However, the BNC was closely related to soil nutrient availability, whereas FNC was linked to the ratio of SOC to total nitrogen. These findings demonstrate that rhizosphere microbial necromass carbon dynamics in response to moisture change are plant-specific and driven by distinct factors. Therefore, accurately projecting future SOC stability in semi-arid regions requires models to incorporate plant-specific traits and microbial mediation.

**Supplementary Information:**

The online version contains supplementary material available at 10.1038/s41598-026-52433-7.

## Introduction

Global climate change has altered precipitation patterns, leading to a rise in the frequency and intensity of precipitation events in semi-arid regions in recent years^1^. Such an increase in soil moisture caused by the increased precipitation can destabilize stored carbon^2^. Soil represents a critical global carbon sink, containing a larger carbon stock than the atmosphere and terrestrial vegetation combined^3^. Soil organic carbon (SOC) serves as a vital indicator of soil health and ecosystem productivity, and is inextricably linked to the functioning of the global carbon cycle^4,5^. Microorganisms mediate SOC dynamics through dual roles: they mineralize organic matter and release CO_2_, while their metabolic byproducts and necromass form stable carbon pools via the ‘entombing effect’^6^. Studies have shown that microbial necromass carbon can contribute up to 80% of SOC, highlighting their importance in carbon sequestration^7,8^. The rhizosphere, as a hotspot of plant-microbe interactions, plays a disproportionately important role in SOC formation and turnover^9^. Different plant species exhibit considerable variation in root exudation and nutrient acquisition strategies, which drive the formation of unique rhizosphere microbiomes and influence carbon dynamics^10,11^. Therefore, investigating how water addition affects the microbial necromass concentrations specifically within the rhizosphere soil is essential to understanding carbon cycling dynamics in semi-arid regions.

The impact of water addition on microbial necromass carbon and their contribution to SOC is complex and context-dependent. Increased moisture can initially stimulate microbial activity and plant growth, potentially boosting necromass production^12,13^. However, excessive moisture may also increase soil microbial nitrogen enzyme activity, which accelerates the decomposition and utilization of microbial necromass due to intensified microbial nitrogen limitation, ultimately reducing the concentration of microbial necromass carbon^14^. Furthermore, microbial groups respond differentially to soil moisture increase. Fungal communities, key contributors to stable necromass, are often more sensitive under high moisture conditions than bacterial communities^15,16^. While existing research has primarily focused on drought effects on microbial necromass carbon in bulk soil^14^, a significant knowledge gap remains regarding how water addition alters the production and stabilization of microbial necromass within the rhizosphere environment, and how this effect is modulated by plant species.

Both bacterial and fungal necromass carbon (BNC and FNC) are regulated by the complex interplay of multiple biotic and abiotic variables^17^. Water addition in semi-arid ecosystems can directly alter soil nutrient availability by stimulating organic matter mineralization and enhancing nutrient diffusion rates^18^. Such changes in nutrient availability, particularly nitrogen and phosphorus, in turn influence microbial metabolic activity and carbon use efficiency^19^. When nutrient availability is high, microorganisms tend to shift their metabolic investment toward anabolism and growth, a strategy that enhances biomass accumulation and ultimately elevates necromass production^6,20^. Under such nutrient‑rich (e.g., increased water) conditions, bacteria are expected to exhibit *r*‑selected life‑history traits due to their short generation times, high metabolic flexibility, and resource opportunism, they rapidly proliferate and contribute to necromass production. In contrast, fungi tend to be more K‑selected or stress‑tolerant^21,22^. Conversely, nutrient limitation can shift metabolism towards respiration, reducing biomass yield^23^. Microbial properties, including microbial biomass and extracellular enzyme activities, are key regulators of microbial necromass carbon accumulation. Soil bacterial and fungal biomass have been found to be correlated with BNC and FNC concentrations, respectively^24^^,25^, while higher soil extracellular enzyme activities can accelerate the turnover and decomposition of necromass^14^. Notably, the FNC is generally more persistent and contributes more to the stable SOC pool than BNC^8,16^. Distinguishing the drivers of BNC and FNC is therefore crucial for predicting their respective roles in SOC stabilization under increased precipitation patterns in semi-arid areas.

The rhizosphere is characterized by enhanced microbial activity and distinct microbial communities due to root exudate inputs, creating a hotspot for necromass formation that is fundamentally different from bulk soil^9,20^. Investigating the rhizosphere soil across different plant types is crucial, as plants modulate the soil environment in species-specific ways^10^. In this study, *Avena sativa* and *Leymus chinensis*, two widely distributed grass species in semi-arid areas, were selected for a three-month indoor pot cultivation experiment. These two species represent distinct life-history strategies. *Avena sativa* (annual) typically allocates more resources to rapid root growth and resource acquisition during its short lifecycle, potentially leading to higher root exudation rates and a more transient rhizosphere effect. In contrast, *Leymus chinensis* (perennial) invests in persistent root systems with slower turnover, which may foster a more stable rhizosphere microbial community and gradual accumulation of organic matter over time^26,27^. Such fundamental differences in root traits are likely to modulate rhizosphere carbon dynamics and microbial necromass accumulation under increased moisture conditions.

This study aimed to quantify how water addition affected microbial necromass carbon in the rhizosphere of these plants, and to elucidate the mechanisms by which soil nutrient availability and microbial properties serve as underlying drivers of necromass carbon accumulation and SOC storage. We hypothesized that (1) microbial necromass carbon would exhibit species-specific responses to water addition, with the perennial *Leymus chinensis* showing increased accumulation due to its persistent root system and stable microbial associations, while the annual *Avena sativa* would show decreased accumulation due to its transient rhizosphere effects; (2) given its greater persistence, variation in FNC would have a stronger impact on SOC concentration than that of BNC; and (3) the BNC would be more responsive to changes in nutrient availability and microbial properties than FNC, because bacteria are generally exhibit more *r*-selected and nutrient-limited than fungi in the rhizosphere^21,22^. This research would elucidate the plant-microbe-soil mechanisms governing SOC sequestration under increased precipitation regimes, providing a scientific basis for predicting carbon dynamics in semi-arid ecosystems.

## Materials and methods

### Study area

The tested soil was collected from Kangbao County, Zhangjiakou City, Hebei Province, China (114° 37′ 12″ E, 41° 51′ 0″ N, 1450 m a.s.l.). The area has a temperate semi-arid continental monsoon climate. The annual average temperature is 2.0 °C, and the annual average precipitation is 350 mm. The soil type is chestnut soil in the Chinese soil classification system, which is equivalent to Kastanozem in the World Reference Base for Soil Resources. At the end of June 2022, soil was collected from the topsoil layer (0–20 cm) of a farmland that had been abandoned for three years. To minimize potential legacy effects from previous plant communities, the soil was air-dried and then homogenized by sieving through a 2-mm mesh to remove surface litter and visible root debris. The collected soil was pre-incubated under controlled conditions for two weeks before the start of the pot experiment to stabilize microbial activity. Key soil physical and chemical properties were determined as follows: pH 8.27, SOC 6.41 g kg^− 1^, total nitrogen (TN) 0.61 g kg^− 1^, and total phosphorus (TP) 40 mg kg^− 1^.

### Experimental design and soil sample collection

On April 1, 2023, *Avena sativa* and *Leymus chinensis*, which are widely grown in semi-arid areas, were selected for an indoor pot culture experiment. Two treatments were set for each plant: control and water addition. In the control treatment, the soil water content was maintained at 30% of its water-holding capacity to simulate the soil water condition in semi-arid areas. In the water addition treatment, the soil water content was maintained at 60% of its water-holding capacity to simulate the soil condition under doubled soil water content. Five replicates were set for each treatment. Seeds were germinated on moist filter paper in Petri dishes and maintained in an incubator at 20 °C for 72 h prior to planting. The germinated seeds were then transplanted into pots measuring 35 cm in upper diameter, 30 cm in lower diameter, and 20 cm in height. Each pot contained about 2.5 kg soil. The pots were weighed with a balance between 9:00 and 10:00 every morning. The weight of the pots was restored to the initial value of the experiment by watering to control the soil water content. In order to ensure the consistency of the experiment, tap water that had been left to stand for one day was used during the incubation. All pots were placed in a culture room and exposed to natural light. On July 1, the rhizosphere soil of each plant was collected following the ‘soil adhering to roots after shaking’ method^28^. A total of 20 rhizosphere soil samples (2 plants × 2 treatments × 5 replicates) were obtained. The fresh rhizosphere soil samples were sieved through a 2-mm mesh prior to subsequent analysis.

### Sample analysis

#### Soil nutrient analysis

The concentration of available nitrogen, including nitrate nitrogen and ammonium nitrogen, was determined by extraction with 2 M KCl (1: 10 soil: solution ratio), followed by quantification on a continuous flow analyzer (Bran Luebbe, AA3, Germany). The concentration of available phosphorus was extracted with 0.5 M NaHCO_3_ solution (1: 20 soil: solution ratio) and analyzed on the same instrument. The concentration of TN was measured by an elemental analyzer (Elementar, Vario Max CN, Germany). The concentration of TP was determined by an inductively coupled plasma-optical emission spectrometry (Optima 5300 DV, Perkin Elmer, USA) following a microwave-assisted digestion (Mars Xpress, CEM, USA) with HNO_3_ and HF. The concentration of SOC was determined using the potassium dichromate oxidation method with external heating. The concentration of particulate organic carbon (POC) was extracted with (NaPO_3_)_6_ solution (5 g L^–1^, 1:5 soil: solution ratio) and then wet-sieved to collect the 53 μm–2 mm fraction, which was then dried and analyzed by an elemental analyzer^29^. The mineral-associated organic carbon (MAOC) concentration was calculated by subtracting the POC concentration from the total SOC^30,31^.

#### DNA extraction and PCR amplification

The concentration of total microbial genomic DNA was extracted from soil samples using the soil DNA kit (E.Z.N.A.^®^soil DNA Kit, Omega Bio-Tek, USA). The extracted DNA was subsequently evaluated by 1.0% agarose gel electrophoresis and a spectrophotometer (NanoDrop2000, Thermo Scientific, USA). The PCR amplification of the bacterial 16S rRNA V3–V4 region was performed with primers 338F/806R (5’- ACTCCTACGGGAGGCAGCAG-3’ and 5’- GGACTACHVGGGTWTCTAAT-3’), while the fungal ITS1 region was amplified using primers ITS1F/ITS2R (5’-CTTGGTCATTTAGAGGAAGTAA-3’ and 5’-GCTGCGTTCTTCATCGATGC-3’) in a PCR thermocycler (T100 Thermal Cycler, BIO-RAD, USA) according to the procedures of Xu et al.^32^ and Adams et al.^33^, respectively. The PCR products were electrophoresed on a 2% agarose gel, then purified with a PCR clean-up kit (C01-10000, Yuhua, China), and finally quantified using a fluorescence quantifier (Qubit 4.0, Thermo Fisher Scientific, USA).

#### Soil extracellular enzyme activities

The potential activities of soil extracellular enzymes, including β−1,4-glucosidase (βG), β−1,4-N-acetylglucosaminidase (NAG), and alkaline phosphomonoesterase (ALP), were determined fluorometrically following Saiya-Cork et al.^34^. The assays were conducted in 96-well microplates by incubating for 4 h at 20 °C in the dark with 50 mM Tris-aminomethane buffer (pH = 8.0) and 400 µM of the respective substrate. Fluorescences were acquired with a microplate fluorometer (Synergy™ H4, BioTek, USA), with the excitation and emission parameters set at 365 nm and 450 nm, respectively. Enzyme activities were quantified by comparison with 4-methylumbelliferone standard curves. To assess microbial resource limitations, the vector length (unit less) and vector angle (◦) of soil extracellular enzyme activities were calculated according to Yang et al.^35^. Vector length reflected the relative carbon vs. nutrient limitation, whereas vector angle indicated relative phosphorus vs. nitrogen limitation.

#### Soil amino sugar concentrations

The amino sugar biomarkers, including muramic acid (MurN) and glucosamine (GluN), were extracted following the procedure established by Appuhn et al.^36^ for the estimation of microbial necromass carbon. Their concentrations were derivatized with orthophthaldialdehyde prior to analysis by a high-performance liquid chromatography (UltiMate 3000, Thermofisher, USA) following the protocol from Indorf et al.^37^. Bacterial necromass carbon (BNC) was estimated directly from MurN concentration. Fungal necromass carbon (FNC) was calculated from fungal-derived GluN, which was obtained by subtracting the bacterial-derived GluN (assumed as twice the MurN concentration) from the total GluN. The calculations of BNC and FNC concentrations specifically referred to Appuhn et al.^38^. Conversion factors of 45 and 9 were used to convert bacterial MurN to BNC and fungal GluN to FNC, respectively^38^. Total microbial necromass carbon was defined as the sum of BNC and FNC concentrations.

### Statistical analysis

All data were confirmed to follow a normal distribution as determined by the Kolmogorov-Smirnov test. Differences in microbial necromass carbon concentrations and their contributions to SOC between treatment groups (control vs. water addition) or plant species were assessed by independent-samples *t*-tests. Linear regression analyses were employed to examine the relationships of BNC, FNC with SOC fractions, nutrient availability, and microbial properties. Above data analyses were conducted in SPSS 18.0 (Chicago, IL, USA) with the significance level set at *p* < 0.05. All figures were generated with SigmaPlot 10.0 (Systat Software Inc., San Jose, USA).

## Results

### The soil nutrient availability

Water addition decreased nitrogen availability, but did not alter phosphorus availability. Specifically, water addition respectively decreased the concentrations of TN and available nitrogen by 8% and 63% under *Avena sativa* rhizosphere, and decreased the concentration of available nitrogen by 46% under *Leymus chinensis* rhizosphere (*p* < 0.05). Additionally, the ratio of SOC/TN in the rhizosphere of *Avena sativa* increased by 1%, and the ratios of SOC/TN and SOC/TP in the rhizosphere of *Avena sativa* respectively increased by 17% and 24% after water addition (*p* < 0.05, Table 1).

**Table 1:** The nutrient availability.

Plant species	Treatments	Total nitrogen (g kg^− 1^)	Available nitrogen (mg kg^− 1^)	Total phosphorus (mg kg^− 1^)	Available phosphorus (mg kg^− 1^)	SOC/TN	SOC/TP	TN/TP
*Avena sativa*	Control	1.04 ± 0.01Aa	17.17 ± 1.36Aa	44.51 ± 2.43Aa	4.12 ± 0.50Ab	6.91 ± 0.05Ba	163.56 ± 8.65Aa	23.63 ± 1.14Aa
	Water addition	0.94 ± 0.01Ba	6.31 ± 0.48Ba	40.18 ± 2.13Aa	4.13 ± 0.47Ab	7.10 ± 0.03Ab	167.83 ± 9.01Aa	23.63 ± 1.24Aa
*Leymus chinensis*	Control	0.98 ± 0.02Ab	10.42 ± 0.71Ab	45.44 ± 2.61Aa	7.85 ± 0.80Aa	6.35 ± 0.17Bb	138.50 ± 7.48Bb	21.78 ± 1.37Ab
	Water addition	0.96 ± 0.02Aa	5.68 ± 0.54Bb	42.42 ± 2.34Aa	9.39 ± 0.84Aa	7.52 ± 0.17Aa	171.80 ± 8.93Aa	22.88 ± 1.30Aa

Values were mean ± standard error. Different uppercase letters indicated significant difference between the two treatments under the same plant species, *p* < 0.05. Different lowercase letters indicated significant difference between the two plant species under the same treatment, *p* < 0.05. SOC/TN = ratio of soil organic carbon to total nitrogen concentration, SOC/TP = ratio of soil organic carbon to total phosphorus concentration, TN/TP = ratio of total nitrogen to total phosphorus concentration.

### The concentrations of soil microbial necromass carbon and SOC

**Fig. 1:**
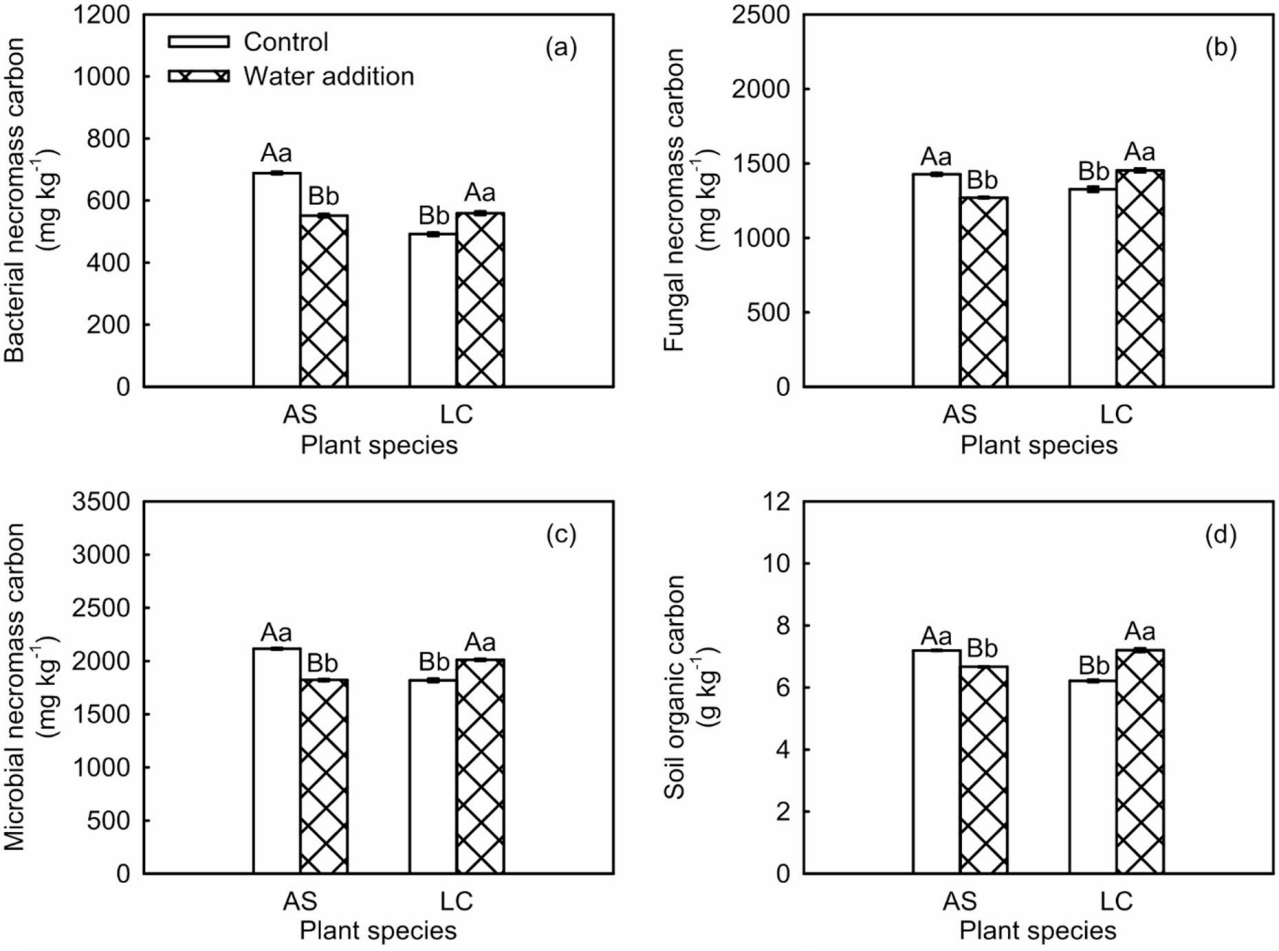
The concentrations of microbial necromass carbon and soil organic carbon. Values were mean ± standard error. Different uppercase letters indicated significant difference between the two treatments under the same plant species, *p* < 0.05. Different lowercase letters indicated significant difference between the two plant species under the same treatment, *p* < 0.05. AS = *Avena sativa*, LC = *Leymus chinensis*.

Water addition decreased the concentrations of BNC, FNC, microbial necromass carbon, and SOC by 20%, 11%, 14%, and 7% under *Avena sativa* rhizosphere, respectively (*p* < 0.05). In contrast, the concentrations of BNC, FNC, microbial necromass carbon, and SOC under *Leymus chinensis* respectively increased by 14%, 9%, 11%, and 16% after water addition (*p* < 0.05). In the control treatment, the concentrations of BNC, FNC, microbial necromass carbon, and SOC in the *Avena sativa* rhizosphere were higher than those in *Leymus chinensis*, while the opposite was observed in the water addition treatment (*p* < 0.05, Fig. 1).

### The contribution of soil microbial necromass carbon to SOC

**Fig. 2:**
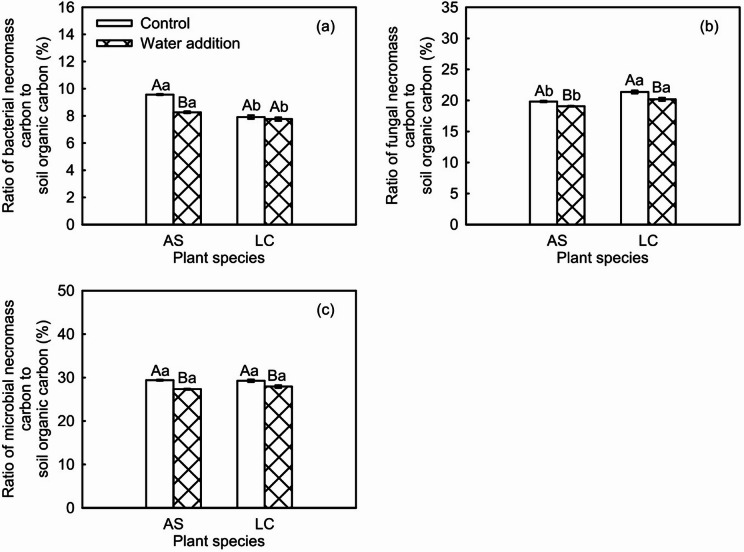
Contribution of microbial necromass carbon to soil organic carbon. Values were mean ± standard error. Different uppercase letters indicated significant difference between the two treatments under the same plant species, *p* < 0.05. Different lowercase letters indicated significant difference between the two plant species under the same treatment, *p* < 0.05. AS = *Avena sativa*, LC = *Leymus chinensis*.

Water addition decreased the contribution of soil microbial necromass carbon to SOC. Specifically, the contribution of BNC, FNC and microbial necromass carbon to SOC respectively decreased by 14%, 4%, and 7%, relative to control under *Avena sativa* rhizosphere after water addition (*p* < 0.05). The contribution of FNC and microbial necromass carbon to SOC respectively decreased by 6% and 5%, relative to control after water addition (*p* < 0.05), while the contribution of BNC to SOC did not change in the *Leymus chinensis* rhizosphere (*p* > 0.05). The contribution of BNC to SOC was higher under *Avena sativa* than those under *Leymus chinensis* in both control and water addition treatments, while the opposite was observed in the contribution of FNC to SOC (*p* < 0.05, Fig. 2).

### Relationships between the concentration of microbial necromass carbon and SOC, nutrient availability, and microbial properties

**Fig. 3:**
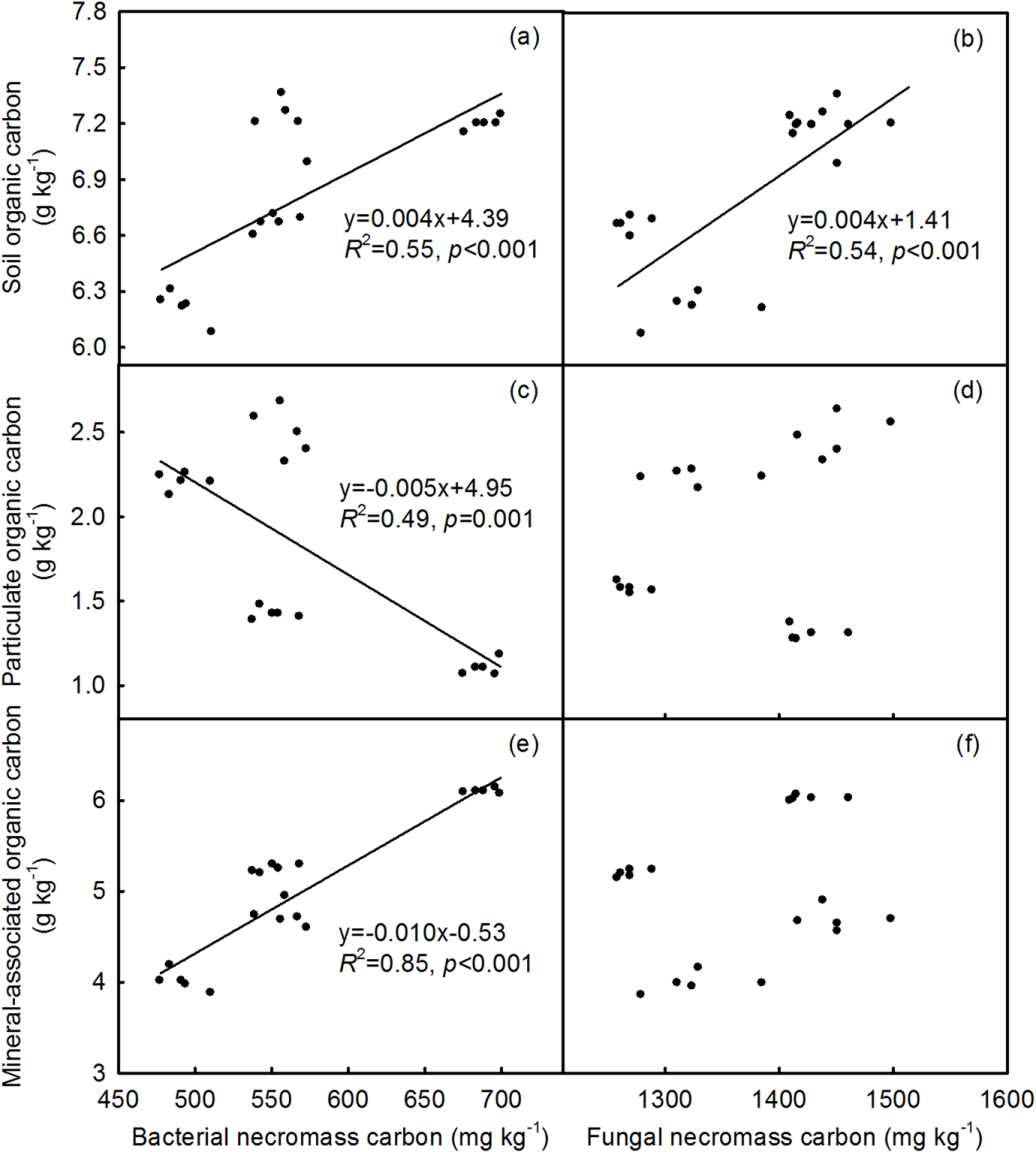
Linear regressions between microbial necromass carbon and soil organic carbon concentrations.

The BNC was positively correlated with SOC and MAOC, and was negatively correlated with POC concentration (*p* < 0.05). This contrasting correlation pattern indicated that BNC is preferentially stabilized in the mineral-associated pool rather than accumulating in the particulate fraction, reflecting its role in forming persistent rather than transient SOC. The FNC was positively correlated with SOC concentration (*p* < 0.05, Fig. 3).

**Fig. 4:**
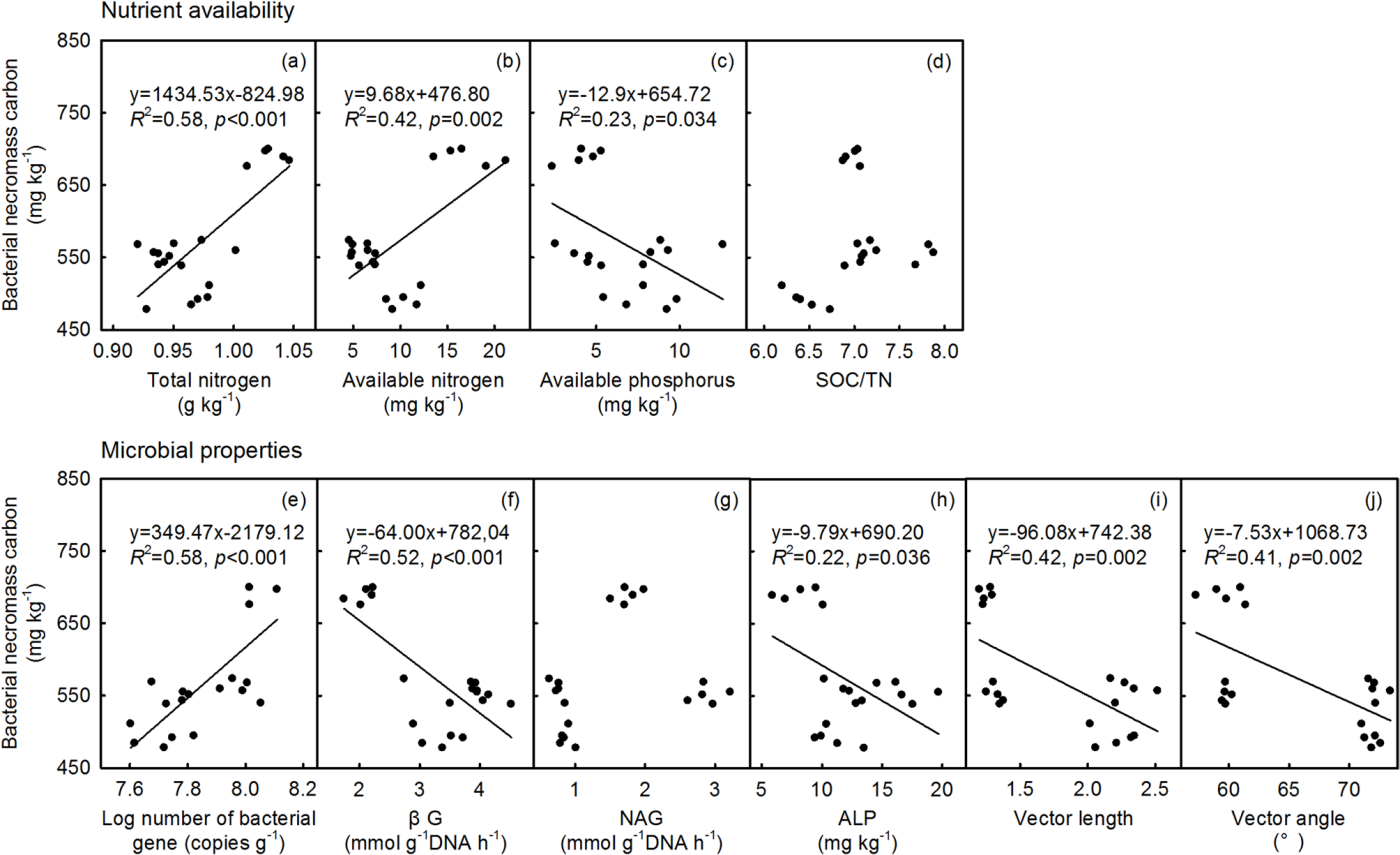
Linear regressions between the concentration of bacterial necromass carbon and nutrient availability, and microbial properties. SOC/TN = ratio of soil organic carbon to total nitrogen, βG = β−1, 4-glucosidase, NAG = β−1, 4-N-acetyglucosaminidase, ALP = alkaline phosphomonesterase.

**Fig. 5:**
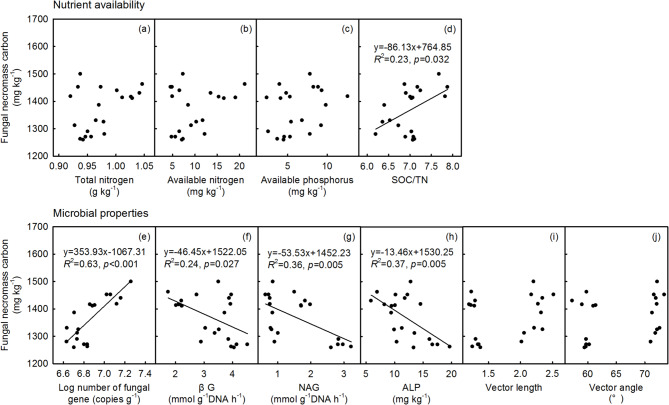
Linear regressions between the concentration of fungal necromass carbon and nutrient availability, and microbial properties. SOC/TN = ratio of soil organic carbon to total nitrogen, βG = β−1, 4-glucosidase, NAG = β−1, 4-N-acetyglucosaminidase, ALP = alkaline phosphomonesterase.

The BNC was positively correlated to TN and available nitrogen concentrations, and bacterial gene abundance, and was negatively correlated to available phosphorus concentration, βG and ALP activities, vector length, and vector angle (*p* < 0.05, Fig. 4 and Fig. S1). The FNC was positively correlated to SOC/TN ratio, and fungal gene abundance, and was negatively correlated to βG, NAG, and ALP activities (*p* < 0.05, Fig. 5 and Fig. S1).

## Discussion

### Influence of water addition on microbial necromass carbon concentration

Consistent with our first hypothesis, the responses of SOC and its components, i.e., BNC and FNC, to water addition diverged in the rhizosphere soils of *Avena sativa* and *Leymus chinensis* (Fig. 1), highlighting the critical role of plant species in steering microbial processes that govern carbon cycling. These results are consistent with the known life-history traits. Fast-growing annuals like *Avena sativa* typically fuel a rapid, short-lived rhizosphere microbiome via high root exudation, which under wet conditions accelerates decomposition. Conversely, slow-growing perennials like *Leymus chinensis* support a more stable, less turnover-prone microbial community through persistent root systems, allowing microbial necromass to accumulate^26,27^.

The increase in both bacterial and fungal gene abundances in the rhizosphere soil of *Leymus chinensis* under water addition (Fig. S2) suggested that improved moisture condition alleviated moisture limitation, supporting a larger and potentially more productive microbial biomass. This aligns with findings that optimal moisture levels can maximize microbial activity and carbon processing^39^. The concomitant rise in the concentrations of BNC, FNC, and SOC indicated that the growing microbial biomass in the rhizosphere of *Leymus chinensis* contributed significantly to necromass formation, which was effectively stabilized in the soil matrix. The specific reduction in NAG activity in the rhizosphere of *Leymus chinensis* was particularly instructive (Fig. S3). The NAG is considered a key enzyme involved in peptidoglycan and chitin degradation, primary components of bacterial and fungal cell walls^40^. Its decreased activity suggested a reduction in the turnover of bacterial and fungal biomass^41^. Thereby, the larger microbial biomass and decreased decomposition of bacterial and fungal necromass likely contributed more necromass without proportionally increasing SOC mineralization, resulting in a net accumulation of soil carbon under *Leymus chinensis*.

In contrast, the rhizosphere of *Avena sativa* exhibited a distinct net loss pattern: both SOC and microbial necromass decreased under water addition. The combination of declined microbial gene abundances and increased enzyme activity (Fig. S2 and Fig. S3) was a hallmark of an ecosystem in a state of high carbon turnover in the rhizosphere of *Avena sativa*. It suggested that the system was prioritizing decomposition over growth, resulting in the net mineralization of existing SOC and microbial biomass through mechanisms such as the ‘priming effect’^42^. This contrasts sharply with the *Leymus chinensis* scenario, where increased microbial biomass and suppressed necromass decomposition drove net carbon accumulation. The concurrent decline in SOC and necromass in *Avena sativa* therefore strengthens the priming argument, indicating that water addition stimulated microbial decomposition of both native SOC and microbial necromass rather than promoting their stabilization.

A pivotal finding here was the decrease in the contribution of FNC to SOC in both plants after water addition (Fig. 2), despite an increase in absolute FNC concentrations in the rhizosphere of *Leymus chinensis* (Fig. 1). One plausible hypothesis to explain this apparent paradox is a ‘dilution effect’. Water addition likely stimulated the input and accumulation of plant-derived organic carbon (such as plant necromass and root exudates) to a greater extent than microbial necromass carbon, thereby reducing the relative contribution of FNC^16,43^. Additionally, increased moisture may favor fast-growing *r*‑strategist fungi that produce less recalcitrant necromass, potentially decoupling fungal abundance from the accumulation of stable FNC^15^. Future studies should combine isotopic labeling (^13^C-CO_2_) or lignin phenol biomarkers to quantify plant-derived carbon inputs, along with fungal community composition analysis, to directly test these mechanisms.

The universal increase in the ratio of fungi to bacteria and the decline in the contribution of FNC to SOC in both plants, appeared paradoxical (Fig. 2 and Fig. S2). This could be because fungal necromass would be less efficiently stabilized in the soil matrix compared to bacterial necromass under new moisture regimes^43^. Bacterial necromass, rich in proteins, may bind more readily to mineral surfaces^6,44^. The change in BNC concentration was more closely related to SOC than FNC (Fig. 3) further supported the notion of a balanced, co-elevation of bacterial biomass production and SOC stabilization under improved soil moisture.

### The contribution of microbial necromass carbon to SOC

The FNC constituted a larger fraction of the total SOC pool (Fig. 2), which was consistent with most studies that also found that fungi contribute more to SOC than bacteria through necromass accumulation in varied ecosystems^16,45^. However, the BNC showed a stronger correlation with SOC concentration than FNC (Fig. 3), which disagreed with our second hypothesis. This apparent paradox highlighted the distinct functional roles and stabilization pathways of fungal and bacterial necromass under the altered soil conditions after water addition. The further observation that BNC was negatively correlated with POC but positively correlated with MAOC (Fig. 3) suggested that bacteria act as efficient processors and direct contributors to the stable carbon pool. Critically, this positive relationship between BNC and MAOC indicated that the newly accumulated BNC under water addition was primarily associated with the MAOC pool rather than the POC pool. Bacteria likely consume the more labile POC, facilitating its transformation through rapid growth and turnover^46^. Their necromass, particularly cell wall fragments rich in proteins and peptidoglycan, possesses chemical properties that favor efficient sorption onto reactive mineral surfaces^6,44^.

Although the experiment lasted only three months, the formation of MAOC via direct adsorption of bacterial necromass-derived compounds onto existing mineral surfaces can occur rapidly (days to weeks), as demonstrated in short-term incubation studies^47^. This rapid abiotic retention does not require the formation of new mineral phases or physical entrapment within macroaggregates, which would indeed take longer. Therefore, the positive relationship between BNC and MAOC likely reflects rapid mineral binding of bacterial necromass to pre-existing mineral surfaces, rather than neo-formation of MAOC through slow aggregation processes. This direct pathway from active processing of POC to the formation of stable MAOC meant that bacterial necromass was closely tied to the contemporary dynamics of carbon input and stabilization, explaining its strong correlation with SOC storage.

Conversely, the lack of significant correlation between FNC and either POC or MAOC underscored its different stabilization mechanism (Fig. 3). Fungal necromass, characterized by recalcitrant compounds like chitin and melanin, is not primarily stabilized through direct mineral association but rather via physical protection within soil aggregates^48,49^. The extensive fungal mycelial network enmeshes soil particles, creating microhabitats where necromass is encapsulated and shielded from decomposition^50^. This process is more dependent on soil structure than on immediate mineral surface chemistry, and it operates on a longer temporal scale^51^. Consequently, over the 3-month experimental timeframe, fungal necromass accumulation did not yet align with the distribution of POC or MAOC pools, as aggregate-mediated physical protection requires more time to become detectable. Thus, the fungal necromass pool represented a large, stable, and slowly cycling background carbon stock, whose dynamics were decoupled from the shorter-term fluctuations of the POC and MAOC pools measured here. These findings provide support for the concept of functional complementarity in the ‘microbial carbon pump’^6^. Bacteria and fungi operate along parallel but distinct pathways: bacteria facilitate a fast channel that processes labile carbon and directly contributes to the MAOC pool, while fungi mediate a slow channel that promotes sequestration through physical protection and aggregate formation. Both pathways are essential for building and maintaining the soil carbon bank. Understanding this dichotomy is crucial for designing management practices that leverage both bacterial and fungal processes to enhance soil carbon sequestration effectively.

### Drivers of microbial necromass carbon under water addition

Partly inconsistent with our third hypothesis, both BNC and FNC showed significant associations with microbial properties. However, the BNC was additionally and more closely related to soil nutrient availability, whereas FNC was additionally and more strongly linked to substrate quality (SOC/TN ratio). Thus, consistent with the general life‑history tendencies in the rhizosphere where bacteria often exhibit more *r*‑selected and nutrient‑limited traits^21,22^, bacterial necromass necromass accumulation is co‑driven by nutrient status and microbial properties. In contrast, fungal necromass is primarily coupled to microbial community traits and substrate quality, with less direct dependence on soil nutrient availability, even though fungi can adopt more *r*‑selected strategies under high‑resource conditions^15^.

The strong positive correlation of BNC and FNC with bacterial and fungal gene abundance, respectively (Figs. 4 and 5), indicated that a larger microbial population directly translated into a greater necromass production potential. Water addition may influence fungal biomass indirectly through its effects on substrate availability and soil microhabitat conditions. Furthermore, the negative relationships between necromass concentrations and enzyme activity (Fig. 5) suggested that while water addition can stimulate overall microbial activity (Fig. S3), the net accumulation of microbial necromass depends on whether that activity is channeled towards decomposition or growth. High enzyme activity indicates active decomposition of necromass itself, which would reduce its net accumulation. When microbial activity is channeled towards growth and turnover rather than aggressive decomposition, it promotes the ‘microbial carbon pump’ and leads to greater necromass stabilization^6,20^.

Beyond these shared microbial drivers, the BNC was additionally and more closely related to soil nutrient availability. The increased soil moisture following water addition likely stimulated organic matter mineralization, leading to higher nitrogen availability in the rhizosphere. The observed decrease in available nitrogen concentration suggested that plant nitrogen uptake outpaced net nitrogen mineralization (Table 1).The BNC showed positive correlations with TN and available nitrogen (Fig. 4), aligning with global evidence that high nitrogen availability promotes BNC accumulation^52^. The minor change in TN relative to the strong decline in available nitrogen (Table 1) reflected the distinct turnover rates of these two pools. The available nitrogen is a small, rapidly cycling pool whose drawdown is driven by increased plant and microbial demand under water addition, whereas TN is a large, recalcitrant reservoir that is insensitive to short-term changes^53^. Under nitrogen-sufficient conditions, bacterial communities could thrive, leading to accelerated turnover and a greater contribution of their necromass to the SOC pool^54,55^. This highlighted the role of nitrogen in fostering bacterial-driven carbon sequestration.

Conversely, the observed negative correlation between BNC and available phosphorus was unexpected (Fig. 4). The vector angles > 45° across all treatments (Fig. S4) indicated persistent phosphorus limitation in both rhizosphere soils^56^. Under such phosphorus limited conditions, microbes typically allocate substantial carbon and energy to synthesizing phosphorus acquisition enzymes rather than to biomass production^57^. Our enzyme activity data supported this allocation pattern. The ALP activity increased with water addition in both species (Fig. S3), indicating that microbial investment in phosphorus acquisition was further upregulated despite the fact that available phosphorus concentration did not increase (Fig. 4). The activity of βG also increased with water addition (Fig. S3), reflecting a concurrent increase in microbial demand for carbon under improved moisture conditions^40^. The response of NAG activity diverged between species. It increased in *Avena sativa* but decreased in *Leymus chinensis* (Fig. S3). This species‑specific pattern suggested that, in *Avena sativa*, water addition stimulated investment in both carbon, nitrogen and phosphorus acquisition enzymes, whereas in *Leymus chinensis*, the reduction in NAG activity may indicate a shift towards more efficient nitrogen cycling or a different microbial community composition.

Importantly, the negative correlation between BNC and available phosphorus persisted even though available phosphorus did not rise with water addition. This suggested that the relationship was not driven by a direct alleviation of phosphorus limitation, but rather by a microbial carbon allocation trade‑off that is deeply rooted in the legacy of phosphorus stress. Under persistent phosphorus limitation (vector angle > 45°), microbes channel a larger fraction of assimilated carbon into extracellular enzyme production at the expense of growth, thereby reducing carbon available for anabolism and subsequent necromass formation^23,58^. The concurrent increase in βG and ALP activities, together with the decrease in BNC under water addition, provided direct enzymatic evidence for this allocation shift, i.e., the microbial community responded to improved moisture not by increasing biomass, but by further investing in nutrient‑acquisition enzymes. Consequently, the negative correlation between BNC and available phosphorus reflected a persistent, legacy‑driven prioritization of enzyme production over growth, rather than a direct inhibitory effect of phosphorus itself or a response to changing phosphorus availability. This interpretation was consistent with the microbial carbon use efficiency framework, where low microbial carbon use efficiency under nutrient limitation leads to reduced biomass synthesis and greater enzyme production^59^.

For FNC, substrate quality emerged as an additional key driver. The positive correlation between FNC and the SOC/TN ratio was particularly insightful. A higher SOC/TN ratio typically indicates a lower quality resource, which favors fungi and their predominantly K-selected strategies under the conditions of this study^60,61^. Fungi are adept at decomposing complex, carbon-rich substrates, and their biomass and necromass become more prominent in such environments^62^. This suggested that substrate quality is an important indirect driver of FNC accumulation, distinguishing it from BNC which is more directly influenced by mineral nutrient availability.

## Conclusions

We revealed the responses of microbial necromass carbon and their contribution to SOC to water addition in the rhizosphere of two plant species in a semi-arid area. The results demonstrated that plant identity was a critical determinant of how soil carbon pools respond to water addition caused by increased precipitation in semi-arid regions. Although water addition enhanced microbial necromass carbon and SOC in the *Leymus chinensis* rhizosphere, it reduced them in the *Avena sativa* rhizosphere. This suggested that replacing perennial plants with annual plants in semi-arid regions may cause the carbon stored in the soil to shift from a sink to a source in a more humid future climate, which would have a direct impact on land management decisions and the predictions of carbon cycle models. Crucially, water addition universally reduced the contribution of microbial necromass carbon to SOC. Furthermore, both BNC and FNC were associated with microbial properties, but their additional drivers diverged: BNC was primarily governed by nitrogen availability, whereas FNC was mainly driven by substrate quality. This decoupling in their formation pathways and stabilization mechanisms highlights the need for plant‑specific and pathway‑differentiated models. Therefore, accurately predicting future soil carbon dynamics under changing precipitation patterns requires integrating specific plant traits as key variables, particularly root lifespan, root exudation rate, and mycorrhizal type (e.g., arbuscular mycorrhizae). These traits directly modulate moisture effects on nutrient availability, microbial community structure, and the balance between microbial necromass production and decomposition.

## Electronic Supplementary Material

Below is the link to the electronic supplementary material.

Supplementary Material 1

## Data Availability

Data will be made available on request. The datasets used and/or analyzed during the current study are available from the corresponding author upon reasonable request.
