# Supplementary material for: Divergent rhizosphere microbial necromass dynamics between two plant species in response to water addition in a semi-arid region
Source: Sci Rep. 2026 May 10;16:21347. doi: 10.1038/s41598-026-52433-7 (PMC13347069; doi:10.1038/s41598-026-52433-7)
Supplement: Supplementary file 1 — Supplementary Material 1 [file 41598_2026_52433_MOESM1_ESM.docx]

Fig. S1 Linear regressions between the concentration of bacterial or fungal necromass carbon and nutrient availability, and microbial properties

Fig. S2 The abundances of bacterial and fungal genes and their ratios in the rhizosphere soil

Values were mean ± standard error. Different uppercase letters indicated significant difference between the two treatments under the same plant species, *p* < 0.05. Different lowercase letters indicated significant difference between the two plant species under the same treatment, *p* < 0.05. AS = *Avena sativa*, LC = *Leymus chinensis*.

Fig. S3 The potential extracellular enzyme activities in the rhizosphere soil

Values were mean ± standard error. Different uppercase letters indicated significant difference between the two treatments under the same plant species, *p* < 0.05. Different lowercase letters indicated significant difference between the two plant species under the same treatment, *p* < 0.05. AS = *Avena sativa*, LC = *Leymus chinensis*.

Fig. S4 The microbial resource limitations in the rhizosphere soil

Values were mean ± standard error. Vector length represented relative carbon vs. nutrient limitation. Vector angles represented relative phosphorus vs. nitrogen limitation, and angles above 45◦ were considered to be relatively more limited by phosphorus than nitrogen. Different uppercase letters indicated significant difference between the two treatments under the same plant species, *p* < 0.05. Different lowercase letters indicated significant difference between the two plant species under the same treatment, *p* < 0.05. AS = *Avena sativa*, LC = *Leymus chinensis*.
